# First Molecular Detection of *Rickettsia conorii* and *Rickettsia helvetica* in Ticks from Dogs in Luxembourg

**DOI:** 10.3390/pathogens14020204

**Published:** 2025-02-19

**Authors:** Guilherme Moreira, Rafaela S. S. Moreira, Floriane André das Neves, Vanessa Swiontek, Patrícia F. Barradas, Sara Gomes-Gonçalves, João R. Mesquita

**Affiliations:** 1 ICBAS—School of Medicine and Biomedical Sciences, Porto University, 4050-313 Porto, Portugal; gmoreiravet@gmail.com (G.M.); rafaelasimaomoreira@gmail.com (R.S.S.M.); info@dorion.lu (V.S.);; 2 EPIUnit—Instituto de Saúde Pública, Universidade do Porto, 4050-600 Porto, Portugal; patricia.barradas@iucs.cespu.pt; 3 Laboratório para a Investigação Integrativa e Translacional em Saúde Populacional (ITR), 4050-600 Porto, Portugal; 4 Department of Sciences, CESPU, CRL, University Institute of Health Sciences (IUCS), 4585-116 Gandra, Portugal; 5 1H-TOXRUN, One Health Toxicology Research Unit, University Institute of Health Sciences, CESPU, CRL, 4585-116 Gandra, Portugal; 6 Centro de Estudos de Ciência Animal (CECA), Instituto de Ciências, Tecnologias e Agroambiente (ICETA), Universidade do Porto (UP), Rua D. Manuel II, Apartado 55142, 4051-401 Porto, Portugal; 7 Associate Laboratory for Animal and Veterinary Science (AL4AnimalS), 1300-477 Lisboa, Portugal

**Keywords:** *Rickettsia conorii*, *Rickettsia helvetica*, vector-borne diseases, public health, Luxembourg

## Abstract

Vector-borne diseases, particularly those caused by *Rickettsia* species, pose a significant public health threat in Europe. Despite extensive research on tick-borne pathogens in various European countries, Luxembourg has yet not been studied for *Rickettsia* spp. in ticks infesting domestic animals. This study aimed to fill this gap by investigating the presence of *Rickettsia* spp. in *Ixodes ricinus* ticks collected from domestic dogs in Luxembourg between April 2023 and April 2024. A total of 61 ticks were examined using molecular techniques, including PCR amplification of the outer membrane protein B (*ompB*), outer membrane protein A (*ompA*), and citrate synthase (*gltA*) genes. Results revealed the presence of *R. helvetica* and *R. conorii* subsp. *raoultii*, with 4.9% of ticks testing positive for *Rickettsia* spp. Phylogenetic analysis confirmed the high genetic identity of the sequences obtained with previously described strains from Europe and Asia. This study highlights the potential risk of emerging tick-borne diseases in Luxembourg and emphasizes the need for ongoing surveillance to better understand the spread of *Rickettsia* spp. in Europe, particularly as climate change may facilitate the expansion of tick populations and their associated pathogens.

## 1. Introduction

Vector-borne diseases represent a significant global health challenge, accounting for over 17% of all infectious diseases and causing more than 700,000 deaths annually. *Rickettsia* bacteria include vector-borne pathogens distributed worldwide, requiring a competent host to survive, with ticks serving as reservoirs for most *Rickettsia* species [1]. Classified as communicable infectious diseases, rickettsioses are anticipated to impose an increasing societal burden due to a combination of factors, including climate change, growing international travel, nature-based recreational activities, and an ageing population [2]. *Rickettsia*, a genus of obligate intracellular bacteria, is primarily transmitted to animals and humans through the bites of arthropod vectors, with Ixodid ticks serving as the main vectors. Other arthropods, including fleas, lice, and mites, also contribute to Rickettsiae transmission [3]. The genus encompasses over 31 recognized species [4], and is classified into four major groups based on phylogenetic and ecological characteristics. These groups include the spotted fever group, which includes species such as the following: *Rickettsia conorii* and *Rickettsia massiliae*; the typhus group, comprising *Rickettsia prowazekii* and *Rickettsia typhi*; the transitional group, which includes *Rickettsia felis* and *Rickettsia akari*; and the ancestral group, represented by species such as *Rickettsia bellii* and *Rickettsia canadensis* [3].

In Eastern Europe, the presence and diversity of *Rickettsia* species in tick populations have been increasingly documented, emphasizing their public health significance. For instance, in Estonia, *R. helvetica* and the recently described Candidatus *R. uralica* have been detected in ticks removed from rodents, marking the first identification of Candidatus *R. uralica* west of the Ural Mountains [5]. Similarly, in Romania, five *Rickettsia* species, including *R. hoogstraalii*, were identified in various tick species, underscoring the need for continuous surveillance due to the potential health risks posed by these pathogens [6]. In Slovakia, a study revealed the presence of *R. raoultii*, *R. slovaca*, *R. helvetica*, and *R. monacensis* in tick populations, with prevalence rates varying significantly depending on the tick species and locality [7]. This diversity of *Rickettsia* spp. is also evident in other parts of Europe. For example, in Northern France, *R. helvetica* was the most prevalent pathogen of a wide range of *Rickettsia* spp. detected in *I. ricinus* ticks, indicating that a broad range of bacteria may contribute to the microbial landscape in the region [8]. In Lithuania, *R. vini* was identified in *I. lividus* ticks collected from sand martin nests, marking the first report of this species in the country [9]. In Northeastern Poland, *R. helvetica*, Candidatus *R. mendelii*, and *R. raoultii* were found in *I. ricinus* and *Dermacentor reticulatus* ticks, confirming the diverse rickettsial landscape in the region [10].

*Rickettsia helvetica* has been identified as a human pathogen, causing symptoms such as fever, rash, meningitis, and carditis. Similarly, *R. conorii*, the causative agent of Mediterranean spotted fever, has been increasingly reported in newly identified subspecies and geographic regions. This expanding distribution highlights a broader epidemiological impact than previously understood, underscoring the need for enhanced surveillance and research into *Rickettsia* spp. as emerging pathogens [11,12,13]. Continuous monitoring and molecular characterization of *Rickettsia* spp. in tick populations are essential for understanding the epidemiology of these pathogens and mitigating the associated health risks [12,14].

To the best of our knowledge, no studies have been conducted to screen ticks infesting domestic animals in Luxembourg. This study aims to fill this gap by specifically investigating the presence of *Rickettsia* species in ticks collected from domestic dogs across the country. Luxembourg was chosen not only due to the lack of baseline data on tick-borne pathogens in domestic animals but also because of the opportunity to collaborate with local veterinary clinics, which facilitated sample collection. This study aims to provide valuable insights into the local epidemiology of *Rickettsia* spp., contributing to a better understanding of their potential impact on animal and public health in the region.

## 2. Materials and Methods

### 2.1. Sampling Site and Sample Collection

A total of 61 ticks were collected from 61 dogs (1 tick per dog) presenting at two veterinary clinics in the southern region of Luxembourg between April 2023 and April 2024. Although ticks were removed from all dogs during their visits to ensure appropriate care, the dogs were selected randomly for the study. There were no exclusion criteria for the selection of the dogs, meaning that all dogs, regardless of age, breed, or health status, were considered for inclusion. To minimize sampling bias and prevent over-representation of individual hosts, only a single tick was collected per dog, ensuring a more balanced assessment of tick-borne *Rickettsia* prevalence across the sampled population. The dogs came in for routine checks and basic veterinary care at the veterinary clinic.

At the first clinic, located in Differdange, 38 ticks were collected. This clinic primarily serves dogs from the southern part of the country but also receives cases from Central Luxembourg and border areas with France and Belgium. The second clinic, located in Rodange, contributed 23 ticks and primarily treats dogs from rural areas in the southern region.

All ticks were collected from owned dogs, the majority of which had been treated for external parasites using various methods, including spot-on treatments, oral tablets, or collars, utilizing a range of active ingredients. To confirm the identification of the tick specimens to the species level, dichotomous keys and reference books on tick biology were used [15,16,17,18].

The dogs presented to the clinic for various purposes. Following physical examination and visual inspection, ticks, if present, were removed using serrated tweezers and placed directly into vials containing 70% ethanol. The specimens were then transferred to the laboratory for analysis.

### 2.2. DNA Extraction and Molecular Characterization

To detect *Rickettsia*, DNA was extracted and processed individually from each of the 61 ticks. Prior to extraction, specimens were decontaminated by immersion in a 10% bleach solution, thoroughly rinsed with deionized water to eliminate residual disinfectant, dried on filter paper, and subsequently placed into 1.5 mL tubes [19].

DNA extraction was performed using a modified QIAamp^®^ DNA Mini Kit (Qiagen, Valencia, CA, USA), following established protocols for nucleic acid isolation from ticks [20]. A sterile scalpel blade was used to create a small ventral incision in each tick, and 420 μL of lysis buffer along with 25 μL of proteinase K solution was added to the corresponding Eppendorf tube. The samples were vortexed for 30 s to ensure proper mixing, centrifuged at 6000× *g* for 2 min, and incubated at 57 °C for 15 min to facilitate lysis. A 350 μL aliquot of the resulting supernatant was transferred to a fresh microcentrifuge tube and mixed with an equivalent volume of RTL buffer. The tubes were vortexed again for 30 s and pulse-centrifuged, and subsequent steps were carried out in accordance with the QIAamp^®^ DNA Mini Kit protocol using an automated QIAcube system (Qiagen^®^ GmbH, Hilden, Germany).

Initial screening for *Rickettsia* DNA was conducted using conventional PCR, targeting a broad-spectrum 511 bp fragment of the outer membrane protein B (*ompB*) gene, as previously described [21]. To validate *ompB*-positive results and genetically characterize *Rickettsia* species, additional PCR assays were performed for a 532 bp fragment of the outer membrane protein A (*ompA*) gene [22] and a near-complete 806 bp fragment of the citrate synthase (*gltA*) gene [23]. The primers utilized in this study are summarized in Table 1.

Three independent PCR assays were performed to amplify the *ompA*, *gltA*, and *ompB* genes. All reactions were carried out using a T100 thermocycler (Bio-Rad, Hercules, CA, USA) with either the Speedy Supreme NZYTaq 2× Green Master Mix (NZYTech, Lisbon, Portugal) or the Xpert Fast Hotstart Mastermix (2×) with dye (GRiSP^®^, Porto, Portugal), following the respective manufacturer’s protocols, in a total reaction volume of 25 μL. Each gene was amplified under specific thermal cycling parameters. The protocol included an initial denaturation at 95 °C for 3 min, followed by 40 cycles of denaturation at 95 °C for 15 s, gene-specific annealing (*ompA*: 56 °C, *gltA*: 58 °C, *ompB*: 54 °C), a brief extension step at 72 °C for 2 s, and a final elongation at 72 °C for 10 min.

Each PCR run incorporated both negative controls (nuclease-free water) and positive controls consisting of previously characterized DNA sequences (Table 2). Amplified products were separated via electrophoresis on individual 1.5% agarose gels stained with Xpert Green Safe DNA gel dye (GRiSP^®^, Porto, Portugal), run at 100 V for 30 min, and subsequently visualized under UV light.

### 2.3. Sequencing and Phylogenetic Analysis

Amplicons of the expected size were purified using the Exo/SAP Go PCR purification kit (Grisp^®^, Porto, Portugal). After purification, bidirectional sequencing was performed using the Sanger dideoxy sequencing method. The resulting sequences were aligned using the BioEdit Sequence Alignment Editor v7.2.3 software package and compared against those available in the NCBI nucleotide database (GenBank, retrieved on 4 December 2024).

For phylogenetic analysis, MEGA-X version 10.2.6 software [25], IQ-TREE2 version 2.3.6 [26], and the Interactive Tree of Life (iTOL) platform [27] were employed. Sequences generated in this study were analysed alongside representative GenBank sequences. The Bayesian Information Criterion (BIC) in IQ-TREE [26] was used to determine the most suitable evolutionary model for each phylogenetic tree, which was then applied in subsequent analyses. Maximum likelihood (ML) bootstrap values were computed using 1000 replicates to assess the robustness of the inferred phylogenies. Sequence editing and multiple alignments were conducted with the BioEdit Sequence Alignment Editor v7.1.9 (Ibis Biosciences), while additional comparisons were performed using the NCBI nucleotide database (https://blast.ncbi.nlm.nih.gov/Blast.cgi, accessed on 25 October 2024).

### 2.4. Statistical Analysis

The occurrence of *Rickettsia* spp. ticks was determined by calculating the proportion of positive samples relative to the total samples analysed, along with a 95% confidence interval (95% CI).

## 3. Results

### 3.1. Morphological Identification of Ticks

From the total of 61 ticks collected, all were identified as *I. ricinus* based on specific morphological features that distinguish it from other closely related species of the genus. These features include long, narrow, and pointed palps, basis capituli which were observed to be rectangular, a distinct trait that differentiates it from species like *I. hexagonus*. The scutum of the specimens was smooth and uniformly reddish-brown, lacking any rough texture or patterns seen in other species. Furthermore, the setae on the bodies were sparse and short, a feature that sets it apart from species such as *I. hexagonus* and *I. canisuga*, which exhibit denser or more robust hair coverage. These morphological characteristics, taken together, provided clear evidence to confirm the identification of the specimens as *I. ricinus* [15,16,17,18].

### 3.2. Identification of Rickettsiae in Examined Ticks

Of the total 61 ticks initially tested for *ompB*, 3 (3/61, 4.9% ( 95% confidence interval [CI]: 0.10–13.7)) tested presumptively positive for *Rickettsia* spp. Further characterization of the *ompB* gene of these three sequences showed two with highest hits with *R. helvetica* and one with *R. conorii*. A *R. helvetica ompB* sequence exhibited 99.8% identity with a *R. helvetica* sequence isolated from *I. ricinus* specimen from Romania (accession no. JX683116). The other *R. helvetica ompB* sequence showed 99.8% identity with a sequence belonging to the same species isolated from a non-specified tick from France (accession no. AF123725). Lastly, the *R. conorii* sequence retrieved showed 98.4% identity with a *R. conorii* sequence isolated from a *D. reticulatus* from Russia (accession no. KU310592).

To confirm positive results by *ompB* gene, ticks were further studied for the *ompA* and *gltA* regions. Both *R. helvetica ompB*-positive samples only yielded amplicons for the *gltA* region while the *R. conorii ompB*-positive sample only yielded amplicon for the *ompA* region.

BLAST analysis of the partial *gltA* gene revealed that one obtained sequence exhibited 100% identity with a *R. helvetica* sequence previously identified in *I. apronophorus* collected in western Siberia, Russia (GenBank accession no. OQ866615), confirming the classification as *R. helvetica*. Similarly, the other *gltA* sequence showed 100% identity with a *R. helvetica* sequence obtained from an unspecified tick in Montana, USA (GenBank accession no. U59723), also confirming the classification as *R. helvetica.*

Blast analyses of the partial *ompA* gene obtained from the *R. conorii ompB*-positive sample showed 100% identity with a *R. conorii* subsp. *raoultii* sequence obtained from a *Dermacentor marginatus* collected in Türkiye (accession no. MK922656), confirming the classification as *R. conorii* subsp. *raoultii*. These results are summarized in Table 3.

**Figure 1:**
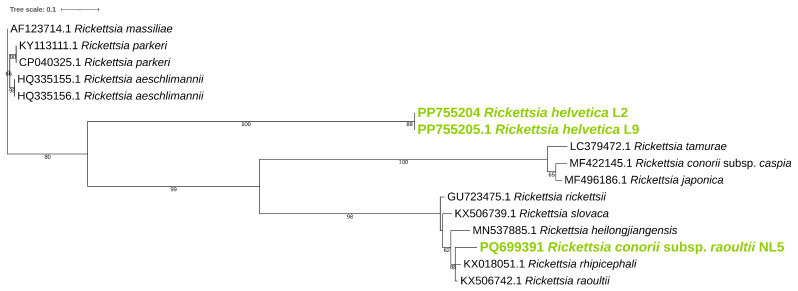
Phylogenetic analysis of the *Rickettsia* spp. identified in ticks. A maximum-likelihood method based on the K3Pu+F+G4 model (Kimura 3-parameter substitution model with unequal base frequencies, incorporating empirical base frequencies derived from the dataset, and accounting for rate heterogeneity among sites using a gamma distribution approximated with four discrete rate categories) phylogenetic tree was constructed based on *Rickettsia ompB* DNA sequences. *Rickettsia* found in this study are highlighted in green.

**Figure 2:**
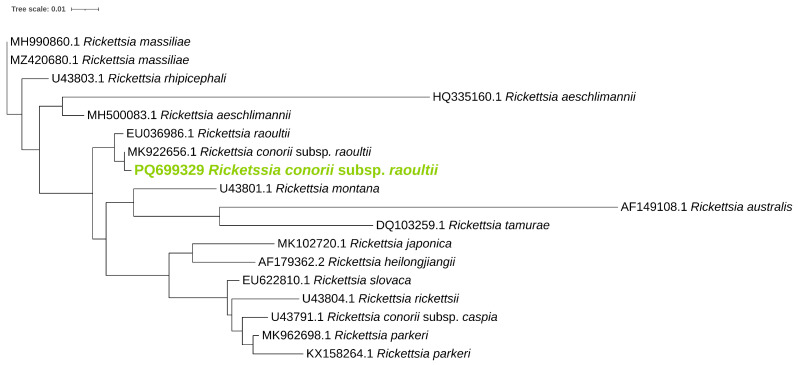
Phylogenetic analysis of the *Rickettsia* spp. identified in ticks. A maximum-likelihood method based on the K3Pu+F+G4 model (Kimura 3-parameter substitution model with unequal base frequencies, incorporating empirical base frequencies derived from the dataset, and accounting for rate heterogeneity among sites using a gamma distribution approximated with four discrete rate categories) phylogenetic tree was constructed based on *Rickettsia ompA* DNA sequences. *Rickettsia* found in this study are highlighted in green.

**Figure 3:**
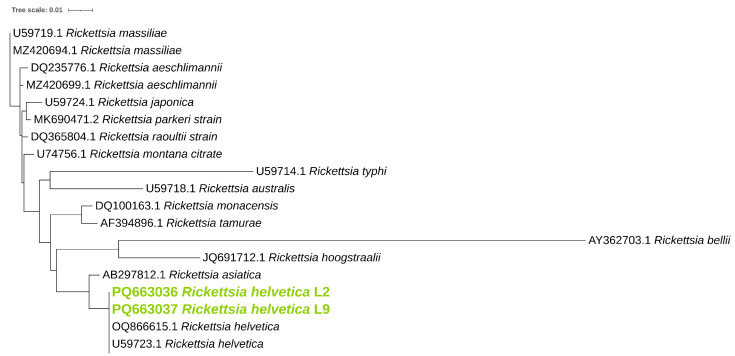
Phylogenetic analysis of the *Rickettsia* spp. identified in ticks. A maximum-likelihood method based on the K3Pu+F+G4 model (Kimura 3-parameter substitution model with unequal base frequencies, incorporating empirical base frequencies derived from the dataset, and accounting for rate heterogeneity among sites using a gamma distribution approximated with four discrete rate categories) phylogenetic tree was constructed based on *Rickettsia gltA* DNA sequences. *Rickettsia* found in this study are highlighted in green.

Phylogenetic analysis was performed for *ompB* (Figure 1), *ompA* (Figure 2), and *gltA* (Figure 3) gene sequences to obtain information regarding their genetic relatedness with other species reference sequences.

## 4. Discussion

*Rickettsia* spp. are vector-borne pathogens with worldwide distribution, including Europe, where they are associated with various tick-borne diseases. Given the knowledge gap in Luxembourg, this study aimed to assess the presence and characteristics of *Rickettsia* spp. in ticks parasitizing dogs. This study identified *R. helvetica* and *R. conorii* subsp. *raoultii* in *I. ricinus* ticks collected from domestic dogs in Luxembourg, with an overall prevalence of 4.9% (95%CI: 0.10-13.7). Molecular characterization using a combination of three primer sets confirmed the presence of these *Rickettsia* species, with positive results from PCR amplification of the *ompB* and *ompA* (for *R. helvetica*), and *ompB* and *gltA* genes (for *R. conorii*). BLAST analysis of the *gltA* gene sequences revealed 100% identity of *R. helvetica* L2 with a strain found in *I. apronophorus* from Western Siberia and of *R. helvetica* L9 with a strain detected in a tick from Montana, USA. This highlights the high genetic conservation and widespread distribution of *R. helvetica*. Similarly, *ompA* sequence analysis showed 100% identity of *R. conorii* subsp. *raoultii* with a strain isolated from *Dermacentor marginatus* ticks in Türkiye. *Rickettsia helvetica* and *R. raoultii* are known to cause various human diseases [12,28]. *R. helvetica* has been associated with fever, meningitis, and carditis [12], while *R. raoultii* has been associated with *Dermacentor*-borne necrosis erythema and lymphadenopathy [29]. *Ixodes* and *Dermacentor* ticks are primary vectors for these pathogens. Their presence in Luxembourg suggests a potential risk for the local population, domestic animals, and wildlife [28,29,30,31]. Furthermore, other studies have detected this species in Europe [32,33], emphasizing its distribution across neighbouring regions and the importance of surveillance to access public health risks.

Discrepancies in PCR results, where either *ompA* or *gltA* amplify but not both, can arise from various factors. Sequence variability or mutations in primer binding sites may hinder amplification, particularly if primers are not optimized for *Rickettsia* strain diversity. Differences in target gene abundance, genomic organization, or DNA degradation during sample collection or storage may preferentially affect one gene. Additionally, natural genomic variability among *Rickettsia* species or strains could result in the absence or low abundance of one gene. Similar discrepancies have been observed in other studies, underscoring the challenges of accurate amplification across diverse bacterial genomes [34]. The limited sample size (*n* = 61) in this study reflects constraints related to the localized scope of Luxembourg, seasonal fluctuations in tick activity, and the specific focus on ticks collected from domestic dogs; while this sample size may limit the generalizability of the findings, the study provides valuable insights into the prevalence of *Rickettsia* species in the region, particularly given the very limited area of the country (2586 km^2^). These results highlight the need for larger-scale studies in the future to improve the robustness of epidemiological assessments and to better understand the distribution and risk of tick-borne pathogens. A limitation of this study is the exclusion of wildlife samples, such as rodents and hedgehogs, which are known to contribute to pathogen maintenance. Future studies should consider including these wildlife reservoirs to provide a more comprehensive understanding of pathogen dynamics [32]. The use of multiple keys ensured robustness and reliability in the identification process, reducing the likelihood of error and increasing confidence in the taxonomic classification of the specimens as belonging to the species *I. ricinus.* Additionally, a ventral incision was made on each tick to facilitate DNA extraction. This approach allows for efficient access to internal tissues where pathogens are typically concentrated. However, as some pathogens may be unevenly distributed within the tick or primarily located in specific tissues not effectively sampled by this method, this could represent a potential limitation of the study.

The identification of *R. helvetica* and *R. conorii* subsp. *raoultii* in Luxembourg is the first report of these species in domestic-dog-associated ticks in the country, highlighting the potential role of companion animals in bridging wildlife reservoirs and human exposure [35]. This trend reflects a broader pattern observed with other *Rickettsia* species, which are increasingly recognized as causative agents of diverse illnesses across the continent. These findings underscore the importance of continued surveillance and research to better understand the epidemiology and clinical impact of *Rickettsia*-associated diseases in Europe [12,29]. The detection of these pathogens in ticks from various regions, including Luxembourg, highlights their potential for further geographical expansion. Such dispersion raises the likelihood of previously unaffected areas becoming hotspots for these diseases, underscoring the need for proactive monitoring and mitigation strategies to address emerging health risks [6,12,28,36]. The role of wildlife, particularly small mammals such as rodents and hedgehogs, is pivotal in the maintenance and dissemination of these pathogens. Acting as reservoir hosts, these animals sustain the transmission cycles of *Rickettsia* spp., thereby contributing to the persistence and potential spread of these pathogens within both natural and urban ecosystems [6,30,37,38]. Lastly, the spread of tick-borne diseases is exacerbated by climate change [39,40]. Rising temperatures increase tick proliferation and extend transmission seasons, expanding the geographic range of these diseases. In Europe, climate change has been linked to a rise in tick-borne diseases, as warming temperatures drive ticks into higher altitudes and latitudes, increasing the risk of these diseases affecting new regions [41,42,43]. Warmer climates enhance tick survival, accelerate their life cycles, and increase their abundance, heightening the risk of disease transmission [44,45]. These shifts underscore the importance of understanding how climatic factors influence tick populations and disease risks [46,47,48]. Climate change is expected to drive ticks into new areas, potentially exposing human and animal populations to tick-borne diseases in regions previously unaffected [38,41,46,49]. These shifts in tick distribution increase the risk of emerging and re-emerging infections, particularly in areas where healthcare systems and veterinary services may be unprepared to diagnose and manage tick-borne diseases. As tick populations expand, so does the likelihood of encountering new *Rickettsia* strains or other vector-borne pathogens, further complicating surveillance and control efforts.

These trends highlight the need for enhanced surveillance and public health preparedness in vulnerable areas [42,43,47,49,50,51]. Strengthening awareness among healthcare professionals and veterinarians is crucial for early detection and timely treatment, reducing the potential burden of severe clinical cases. Additionally, public education initiatives on tick-bite prevention, responsible pet management, and habitat modifications can help mitigate the risk of exposure. Integrating multidisciplinary approaches that involve epidemiologists, veterinarians, ecologists, and policymakers will be essential in developing effective tick control measures and safeguarding both human and animal health.

In conclusion, the detection of *R. helvetica* and *R. conorii* subsp. *raoultii* in ticks collected from Luxembourg highlights the expanding threat of tick-borne rickettsioses in Europe. These findings, together with the identification of these pathogens in a variety of geographically distant locations, suggest that their distribution may be widening. Climate change may be playing a significant role in reshaping tick populations, enhancing their survival rates, and extending their active periods, which in turn increases the potential for disease transmission. As ticks move into new, previously unsuitable regions—at higher altitudes and latitudes—the risk of these diseases spreading to areas that were not previously affected rises. This underscores the urgent need for ongoing surveillance and effective public health measures to address these evolving threats. A better understanding of how climate change influences tick distribution and pathogen dynamics will be essential for mitigating the risk of emerging tick-borne diseases. Predictive models and early detection systems will play a key role in anticipating shifts in disease transmission, guiding targeted interventions, and helping prioritize resources in the areas that are at the greatest risk. Continued research and monitoring remain crucial in preparing for the future public health challenges posed by climate-driven changes in tick ecology.

## Figures and Tables

**Table 1:** Primers used for PCR amplification of the *ompB*, *ompA*, and *gltA* genes.

Gene	Primer	Nucleotide Sequence (5′–3′)
*ompB*	*ompB F*	GTAACCGGAAGTAATCGTTTCGTAA
	*ompB R*	GCTTTATAACCAGCTAAACCACC
*ompA*	*ompA F*	ATGGCGAATATTTCTCCAAAA
	*ompA R*	AGTGCAGCATTCGCTCCCCCT
*gltA*	*gltA F*	GCTATTATGCTTGCGGCTGT
	*gltA R*	TGCATTTCTTTCCATTGTGC

**Table 2:** Accession numbers of positive controls used for PCR amplification.

Gene	Accession Number	Citation
*ompA*	MZ420682	[24]
*gltA*	MZ420697	[24]
*ompB*	MZ420689	[24]

**Table 3:** Summary of GenBank accession numbers, closest BLAST matches, and identity percentages for the *ompB, ompA*, and *gltA* genes from tick samples L2, L9, and Nl5. Sequences for *Rickettsia* spp. were identified and characterized, with the closest matches corresponding to *R. helvetica* and *R. conorii*.

Gene	Sample	Accession Number	Closest Match	Identity (%)
*ompB*	L2	PP755204	*R. helvetica* (JX683116)	99.8%
	L9	PP755205	*R. helvetica* (AF123725)	99.8%
	NL5	PQ699391	*R. conorii* (KU310592)	98.4%
*ompA*	NL5	PQ699392	*R. conorii* subsp. *raoultii* (MK922656)	100%
*gltA*	L2	PQ66306	*R. helvetica* (OQ866615)	100%
	L9	PQ66306	*R. helvetica* (U59723)	100%

## Data Availability

The data presented in this study are available on request from the corresponding author.

## References

[1] Vector-Borne Diseases. https://www.who.int/news-room/fact-sheets/detail/vector-borne-diseases.

[2] CDC Vector-Borne Diseases. https://www.cdc.gov/climate-health/php/effects/vectors.html.

[3] Blanton L.S. (2019). The Rickettsioses: A Practical Update. Infect. Dis. Clin. N. Am..

[4] Parte A.C. (2014). LPSN—List of prokaryotic names with standing in nomenclature. Nucleic Acids Res..

[5] Vikentjeva M., Geller J., Remm J., Golovljova I. (2021). *Rickettsia* spp. in rodent-attached ticks in Estonia and first evidence of spotted fever group Rickettsia species *Candidatus Rickettsia uralica* in Europe. Parasites Vectors.

[6] Ivan T., Matei I., Novac C., Kalmár Z., Borșan S.D., Panait L., Gherman C., Ionică A., Papuc I., Mihalca A. (2022). Spotted Fever Group Rickettsia spp. Diversity in Ticks and the First Report of *Rickettsia hoogstraalii* in Romania. Vet. Sci..

[7] Špitalská E., Sparagano O., Stanko M., Schwarzová K., Špitalský Z., Škultéty L., Havlíková S.F. (2018). Diversity of *Coxiella*-like and *Francisella*-like endosymbionts, and *Rickettsia* spp., *Coxiella burnetii* as pathogens in the tick populations of Slovakia, Central Europe. Ticks Tick-Borne Dis..

[8] Bonnet S.I., Paul R.E.L., Bischoff E., Cote M., Naour E.L. (2017). First identification of *Rickettsia helvetica* in questing ticks from a French Northern Brittany Forest. PLoS Neglected Trop. Dis..

[9] Matulaitytė V., Paulauskas A., Bratchikov M., Radzijevskaja J. (2020). New record of Rickettsia vini in *Ixodes lividus* ticks from Lithuania. Ticks Tick-Borne Dis..

[10] Stańczak J., Biernat B., Racewicz M., Zalewska M., Matyjasek A. (2017). Prevalence of different *Rickettsia* spp. in *Ixodes ricinus* and *Dermacentor* reticulatus ticks (Acari: Ixodidae) in north-eastern Poland. Ticks Tick-Borne Dis..

[11] Blanco J., Oteo J. (2006). Rickettsiosis in Europe. Ann. N. Y. Acad. Sci..

[12] Oteo J.A., Portillo A. (2012). Tick-borne rickettsioses in Europe. Ticks Tick-Borne Dis..

[13] Portillo A., Santibáñez S., García-Álvarez L., Palomar A., Oteo J. (2015). Rickettsioses in Europe. Microbes Infect..

[14] Hildebrandt A., Krämer A., Sachse S., Straube E. (2010). Detection of *Rickettsia* spp. and *Anaplasma phagocytophilum* in *Ixodes ricinus* ticks in a region of Middle Germany (Thuringia). Ticks Tick-Borne Dis..

[15] Key to Genera | ESCCAP UK & Ireland. https://www.esccapuk.org.uk/page/Key+to+Genera/49/.

[16] Bugmyrin S., Belova O., Bespyatova L.A., Ieshko E.P., Karganova G. (2016). Morphological features of Ixodes persulcatus and *I. ricinus* hybrids: Nymphs and adults. Exp. Appl. Acarol..

[17] Saracho-Bottero M.N., Venzal J., Tarragona E.L., Thompson C., Mangold A., Beati L., Guglielmone A., Nava S. (2019). The *Ixodes ricinus* complex (Acari: Ixodidae) in the Southern Cone of America: *Ixodes pararicinus*, *Ixodes aragaoi*, and *Ixodes* sp. cf. *I. affinis*. Parasitol. Res..

[18] Sonenshine D.E., Roe R.M. (2014). Biology of Ticks. Volume 1.

[19] Sayler K.A., Wamsley H.L., Pate M., Barbet A.F., Alleman A.R. (2014). Cultivation of *Rickettsia amblyommii* in tick cells, prevalence in Florida lone star ticks (*Amblyomma americanum*). Parasites Vectors.

[20] Crowder C.D., Rounds M.A., Phillipson C.A., Picuri J.M., Matthews H.E., Halverson J., Schutzer S.E., Ecker D.J., Eshoo M.W. (2010). Extraction of total nucleic acids from ticks for the detection of bacterial and viral pathogens. J. Med. Entomol..

[21] Choi Y.J., Lee S.H., Park K.H., Koh Y.S., Lee K.H., Baik H.S., Choi M.S., Kim I.S., Jang W.J. (2005). Evaluation of PCR-Based Assay for Diagnosis of Spotted Fever Group Rickettsiosis in Human Serum Samples. Clin. Diagn. Lab. Immunol..

[22] Regnery R.L., Spruill C.L., Plikaytis B.D. (1991). Genotypic identification of rickettsiae and estimation of intraspecies sequence divergence for portions of two rickettsial genes. J. Bacteriol..

[23] De Sousa R., Ismail N., Dória-Nóbrega S., Costa P., Abreu T., França A., Amaro M., Proença P., Brito P., Poças J. (2005). The Presence of Eschars, but Not Greater Severity, in Portuguese Patients Infected with Israeli Spotted Fever. Ann. N. Y. Acad. Sci..

[24] Santos-Silva S., Santos N., Boratyński Z., Mesquita J.R., Barradas P.F. (2023). Diversity of *Rickettsia* spp. in ticks from wild mammals of Morocco and Mauritania. Ticks Tick-Borne Dis..

[25] Kumar S., Stecher G., Li M., Knyaz C., Tamura K. (2018). MEGA X: Molecular Evolutionary Genetics Analysis across Computing Platforms. Mol. Biol. Evol..

[26] Minh B.Q., Schmidt H.A., Chernomor O., Schrempf D., Woodhams M.D., von Haeseler A., Lanfear R. (2020). IQ-TREE 2: New Models and Efficient Methods for Phylogenetic Inference in the Genomic Era. Mol. Biol. Evol..

[27] Letunic I., Bork P. (2019). Interactive Tree Of Life (iTOL) v4: Recent updates and new developments. Nucleic Acids Res..

[28] Sprong H., Wielinga P., Fonville M., Reusken C., Brandenburg A., Borgsteede F., Gaasenbeek C., Giessen J.V.D.v.d. (2009). *I. ricinus* ticks are reservoir hosts for Rickettsia helvetica and potentially carry flea-borne Rickettsia species. Parasites Vectors.

[29] Rudolf I., Venclíková K., Blažejová H., Betášová L., Mendel J., Hubálek Z., Parola P. (2016). First report of *Rickettsia raoultii* and *Rickettsia helvetica* in *Dermacentor reticulatus* ticks from the Czech Republic. Ticks Tick-Borne Dis..

[30] Biernat B., Stańczak J., Michalik J., Sikora B., Wierzbicka A. (2016). Prevalence of infection with *Rickettsia helvetica* in *Ixodes ricinus* ticks feeding on non-rickettsiemic rodent hosts in sylvatic habitats of west-central Poland. Ticks Tick-Borne Dis..

[31] Mediannikov O., Matsumoto K., Samoylenko I., Drancourt M., Roux V., Rydkina E., Davoust B., Tarasevich I., Brouqui P., Fournier P. (2008). *Rickettsia raoultii* sp. nov., a spotted fever group rickettsia associated with Dermacentor ticks in Europe and Russia. Int. J. Syst. Evol. Microbiol..

[32] Jahfari S., Ruyts S.C., Frazer-Mendelewska E., Jaarsma R., Verheyen K., Sprong H. (2017). Melting pot of tick-borne zoonoses: The European hedgehog contributes to the maintenance of various tick-borne diseases in natural cycles urban and suburban areas. Parasites Vectors.

[33] Szekeres S., Docters van Leeuwen A., Tóth E., Majoros G., Sprong H., Földvári G. (2019). Road-killed mammals provide insight into tick-borne bacterial pathogen communities within urban habitats. Transbound. Emerg. Dis..

[34] Miranda J., Mattar S. (2014). Molecular detection of *Rickettsia bellii* and *Rickettsia* sp. strain *Colombianensi* in ticks from Cordoba, Colombia. Ticks Tick-Borne Dis..

[35] Parola P. (2004). Tick-borne rickettsial diseases: Emerging risks in Europe. Comp. Immunol. Microbiol. Infect. Dis..

[36] Grassi L., Menandro M.L., Cassini R., Mondin A., Pasotto D., Grillini M., Rocca G., Drigo M. (2022). High Prevalence of Tick-Borne Zoonotic *Rickettsia slovaca* in Ticks from Wild Boars, Northeastern Italy. Animals.

[37] Speck S., Perseke L., Petney T., Skuballa J., Pfäffle M., Taraschewski H., Bunnell T., Essbauer S., Dobler G. (2013). Detection of *Rickettsia helvetica* in ticks collected from European hedgehogs (*Erinaceus europaeus*, Linnaeus, 1758). Ticks Tick-Borne Dis..

[38] Sréter T., Lancz Z.S., Széll Z., Egyed L. (2005). [*Rickettsia helvetica*: An emerging tick-borne pathogen in Hungary and Europe]. Orvosi Hetil..

[39] Zhang L., Ma D., Li C., Zhou R., Wang J., Liu Q. (2022). Projecting the Potential Distribution Areas of *Ixodes scapularis* (Acari: Ixodidae) Driven by Climate Change. Biology.

[40] Yang X., Gao Z., Wang L., Xiao L., Dong N., Wu H., Li S. (2021). Projecting the potential distribution of ticks in China under climate and land use change. Int. J. Parasitol..

[41] Alkishe A., Raghavan R., Peterson A. (2021). Likely Geographic Distributional Shifts among Medically Important Tick Species and Tick-Associated Diseases under Climate Change in North America: A Review. Insects.

[42] Voyiatzaki C., Papailia S.I., Venetikou M., Pouris J., Tsoumani M., Papageorgiou E. (2022). Climate Changes Exacerbate the Spread of *Ixodes ricinus* and the Occurrence of Lyme Borreliosis and Tick-Borne Encephalitis in Europe—How Climate Models Are Used as a Risk Assessment Approach for Tick-Borne Diseases. Int. J. Environ. Res. Public Health.

[43] Lee J.S., Chung S.Y. (2022). The threat of climate change on tick-borne infections: Rising trend of infections and geographical distribution of climate risk factors associated with ticks. J. Infect. Dis..

[44] Lindgren E., Talleklint L., Polfeldt T. (1999). Impact of climatic change on the northern latitude limit and population density of the disease-transmitting European tick *Ixodes ricinus*. Environ. Health Perspect..

[45] Porretta D., Mastrantonio V., Amendolia S., Gaiarsa S., Epis S., Genchi C., Bandi C., Otranto D., Urbanelli S. (2013). Effects of global changes on the climatic niche of the tick *Ixodes ricinus* inferred by species distribution modelling. Parasites Vectors.

[46] Estrada-Peña A., Ayllón N., Fuente J.D.L. (2012). Impact of Climate Trends on Tick-Borne Pathogen Transmission. Front. Physiol..

[47] Gilbert L. (2009). Altitudinal patterns of tick and host abundance: A potential role for climate change in regulating tick-borne diseases?. Oecologia.

[48] Ogden N., Radojević M., Wu X., Duvvuri V., Leighton P., Wu J. (2014). Estimated Effects of Projected Climate Change on the Basic Reproductive Number of the Lyme Disease Vector *Ixodes scapularis*. Environ. Health Perspect..

[49] Semenza J., Suk J.E. (2017). Vector-borne diseases and climate change: A European perspective. FEMS Microbiol. Lett..

[50] Ogden N., Ogden N., Beard C., Ginsberg H., Tsao J. (2020). Possible Effects of Climate Change on Ixodid Ticks and the Pathogens They Transmit: Predictions and Observations. J. Med. Entomol..

[51] Ma R., Li C., Gao A., Jiang N., Li J., Hu W., Feng X. (2024). Tick species diversity and potential distribution alternation of dominant ticks under different climate scenarios in Xinjiang, China. PLoS Neglected Trop. Dis..

